# *Sordaria macrospora*: 25 years as a model organism for studying the molecular mechanisms of fruiting body development

**DOI:** 10.1007/s00253-020-10504-3

**Published:** 2020-03-11

**Authors:** Ines Teichert, Stefanie Pöggeler, Minou Nowrousian

**Affiliations:** 1 General and Molecular Botany, Ruhr-University Bochum, 44780 Bochum, Germany; 2 Institute of Microbiology and Genetics, Department of Genetics of Eukaryotic Microorganisms, Georg-August University, Göttingen, Germany; 3 Department of Molecular and Cellular Botany, Ruhr-University Bochum, ND 7/176 Universitätsstr. 150, 44780 Bochum, Germany

**Keywords:** *Sordaria macrospora*, Multicellular development, Fruiting body, Sexual development, Analysis of mutants, Genomics, Transcriptomics, Autophagy, RNA editing

## Abstract

**Abstract:**

Fruiting bodies are among the most complex multicellular structures formed by fungi, and the molecular mechanisms that regulate their development are far from understood. However, studies with a number of fungal model organisms have started to shed light on this developmental process. One of these model organisms is *Sordaria macrospora*, a filamentous ascomycete from the order *Sordariales*. This fungus has been a genetic model organism since the 1950s, but its career as a model organism for molecular genetics really took off in the 1990s, when the establishment of a transformation protocol, a mutant collection, and an indexed cosmid library provided the methods and resources to start revealing the molecular mechanisms of fruiting body development. In the 2000s, “omics” methods were added to the *S. macrospora* tool box, and by 2020, 58 developmental genes have been identified in this fungus. This review gives a brief overview of major method developments for *S. macrospora*, and then focuses on recent results characterizing different processes involved in regulating development including several regulatory protein complexes, autophagy, transcriptional and chromatin regulation, and RNA editing.

**Key points:**

•*Sordaria macrospora is a model system for analyzing fungal fruiting body development.*

•*More than 100 developmental mutants are available for S. macrospora.*

•*More than 50 developmental genes have been characterized in S. macrospora.*

## Introduction

**Fig. 1:**
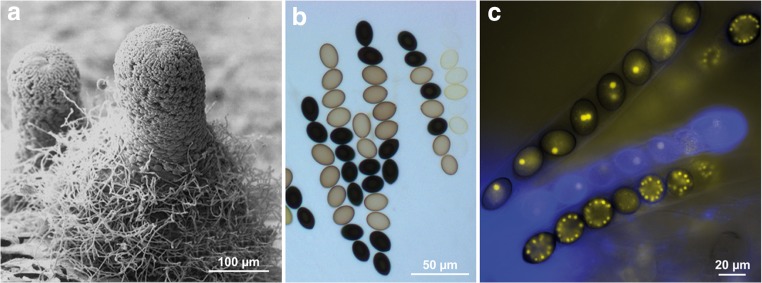
Fruiting bodies and asci of *S. macrospora*. **a** Scanning electron micrograph of a fruiting body (perithecium). **b** Light microscopy of asci from a cross between the wild type (black spores) and a spore color mutant with yellow spores. **c** Fluorescence microscopy of asci and ascopores. Nuclei are labeled with YFP-tagged histones. In the upper ascus, each ascospore contains one to two nuclei. In the lower ascus, each ascospore contains several nuclei after several mitotic divisions. Pictures from Pöggeler et al. (2018) with permission by Springer Nature

**Fig. 2:**
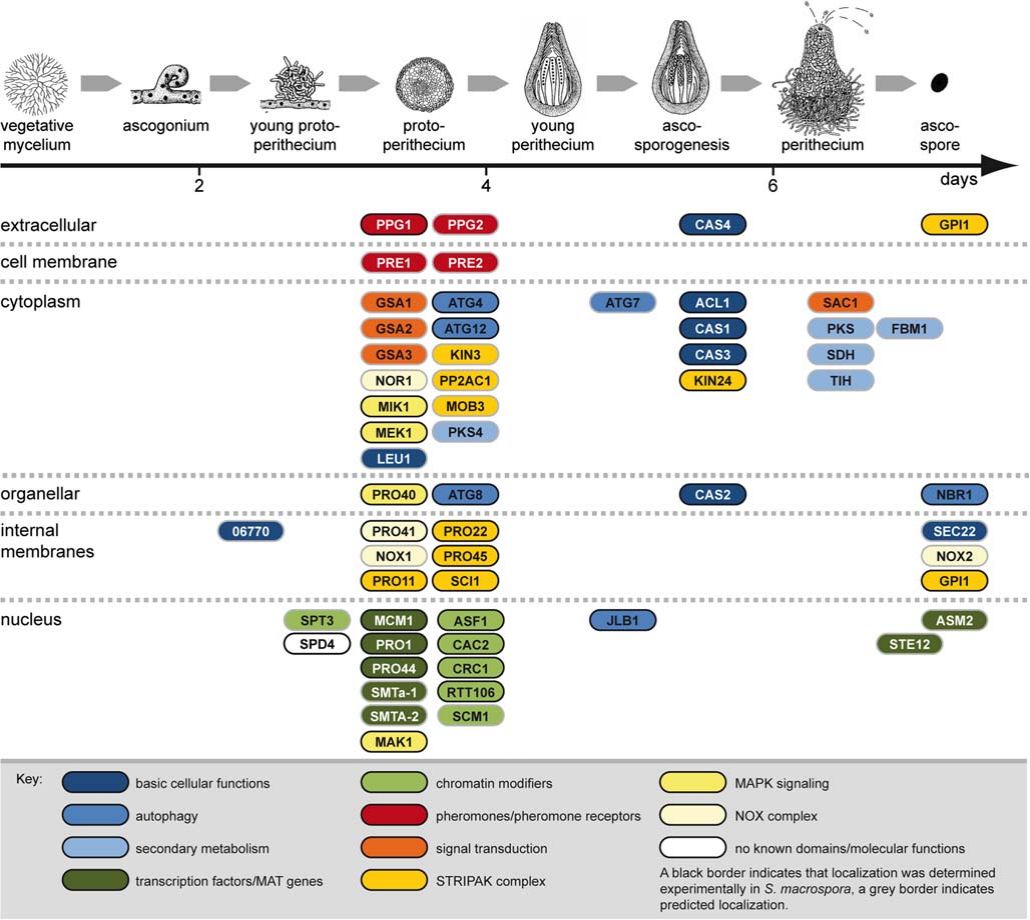
Overview of developmental proteins from *S. macrospora*. Proteins are sorted vertically according to their subcellular localization (indicated on the left), and horizontally according to the developmental stage during which they are required (indicated on top) based on the phenotypes of the corresponding mutants. Pictures of life cycle stages from Kück et al. (2009) with permission by Springer Nature

*Sordaria macrospora* is a filamentous ascomycete with a decades-long history as a model organism to study fruiting body development, one of the most complex forms of multicellular development in fungi (Kück et al. 2009; Engh et al. 2010; Teichert et al. 2014a; Nagy et al. 2018). One reason for its success as a model organism is its short life cycle, which takes only 7 days to be completed under laboratory conditions. Furthermore, *S. macrospora* is homothallic, i.e., self-fertile, which means that a single strain can complete the life cycle without the need of a mating partner. Thus, after inoculation on a Petri dish, no further steps (e.g., fertilization) are required to complete the sexual cycle, which leads to the production of meiotic spores (ascospores) within fruiting bodies (Fig. 1, Fig. 2). Ascospores are the only spores produced by *S. macrospora*, since this fungus lacks any vegetative sporulation. This lack of vegetative spores facilitates handling in the laboratory, because there is less danger of contamination. In recent years, the lack of vegetative spores turned out to have other benefits, namely in transcriptomics studies, because gene expression changes during the sexual cycle are not confounded by other developmental processes running in parallel (Teichert et al. 2014a).

Despite being homothallic, *S. macrospora* is also able to outcross; therefore different strains, e.g., developmental mutants, can be crossed in the laboratory (Esser and Straub 1956). For such classical genetic analyses, another advantage of *S. macrospora* is the production of its ascospores as ordered tetrads, which means that each ascus (meiosporangium) contains the four meiotic products of a single meiosis, and that the order of spores in the ascus allows distinguishing between alleles that segregated in the first versus the second meiotic division (Kück et al. 2009). Especially, the ordered tetrads make *S. macrospora* also a model organism for studying the molecular mechanisms of meiosis. This was reviewed recently by Zickler and Espagne (2016), whereas the focus of this review will be on the use of *S. macrospora* as a model organism for fruiting body development.

The first studies of fruiting body development in *S. macrospora* were conducted in the 1950s using X-ray mutagenesis and genetic crosses (Esser and Straub 1958). However, *S. macrospora* truly began its career as a model organism for fruiting body development with its entry into the world of molecular techniques 25 years ago, starting with the establishment of a transformation system by Walz and Kück (1995). Shortly afterwards, two important resources were developed for *S. macrospora*, namely a mutant collection of more than 100 developmental mutants generated by chemical or UV mutagenesis, and an indexed cosmid library that could be used to transform mutants to fertility, thereby identifying the defective genes in the mutant strains (Pöggeler et al. 1997; Masloff et al. 1999; Kück et al. 2009). In the following years, the developmental genes *acl1*, *pro1*, *pro11*, *pro22*, *pro40*, and *pro41* were identified by complementing sterile mutant strains with an indexed cosmid library (Masloff et al. 1999; Nowrousian et al. 1999; Pöggeler and Kück 2004; Engh et al. 2007b; Nowrousian et al. 2007; Bloemendal et al. 2010). Subsequent analyses of the functions of the corresponding proteins revealed that most of these genes encode members of conserved protein complexes involved in developmental signal transduction, thereby showing the value of a mutant collection with distinct developmental phenotypes (Table 1, Fig. 2).

**Table 1:** Developmental genes from *S. macrospora*. References: [1] (Masloff et al. 1999), [2] (Nowrousian et al. 1999), [3] (Pöggeler and Kück 2004), [4] (Kück 2005), [5] (Mayrhofer and Pöggeler 2005), [6] (Mayrhofer et al. 2006), [7] (Nolting and Pöggeler 2006a), [8] (Nolting and Pöggeler 2006b), [9] (Pöggeler et al. 2006), [10] (Engh et al. 2007a), [11] (Engh et al. 2007b), [12] (Nowrousian et al. 2007), [13] (Kamerewerd et al. 2008), [14] (Elleuche and Pöggeler 2009), [15] (Nolting et al. 2009), [16] (Nowrousian 2009), [17] (Bloemendal et al. 2010), [18] (Klix et al. 2010), [19] (Bernhards and Pöggeler 2011), [20] (Bloemendal et al. 2012), [21] (Gesing et al. 2012), [22] (Nowrousian et al. 2012), [23] (Voigt and Pöggeler 2013a), [24] (Voigt et al. 2013), [25] (Dirschnabel et al. 2014), [26] (Lehneck et al. 2014), [27] (Schindler and Nowrousian 2014), [28] (Teichert et al. 2014b), [29] (Frey et al. 2015a), [30] (Frey et al. 2015b), [31] (Nordzieke et al. 2015), [32] (Traeger and Nowrousian 2015), [33] (Beier et al. 2016), [34] (Werner et al. 2016), [35] (Teichert et al. 2017b), [36] (Radchenko et al. 2018), [37] (Reschka et al. 2018), [38] (Schumacher et al. 2018), [39] (Lütkenhaus et al. 2019), [40] (Werner et al. 2019)

Gene	Locus tag number	Gene product/conserved domains	Ref.
Primary metabolism and basic cellular functions			
*acl1*	*SMAC_06775*	Subunit of the ATP citrate lyase	[2]
*cas1*	*SMAC_03420*	β-class carbonic anhydrase	[14, 26]
*cas2*	*SMAC_03614*	β-class carbonic anhydrase	[14, 26]
*cas3*	*SMAC_03179*	β-class carbonic anhydrase	[14, 26]
*cas4*	*SMAC_03821*	α-class carbonic anhydrase	[26]
*leu1*	*SMAC_07802*	β-isopropylmalate dehydrogenase	[4]
*sec22*	*SMAC_06625*	SNARE protein	[32]
--	*SMAC_06770*	Putative glycolipid 2-alpha-mannosyltransferase	[39]
Autophagy genes			
*Smatg4*	*SMAC_08321*	Cysteine protease	[23]
*Smatg7*	*SMAC_06539*	E1-like enzyme	[15]
*Smatg8*	*SMAC_02305*	Ubiquitin-like protein	[23]
*Smatg12*	*SMAC_06998*	Ubiquitin-like protein	[34]
*Smjlb1*	*SMAC_08510*	bZIP transcription factor	[24]
*Smnbr1*	*SMAC_07844*	Autophagy cargo receptor	[40]
Secondary metabolism			
*fbm1*	*SMAC_00522*	Monooxygenase	[16]
*pks4*	*SMAC_05695*	Reducing polyketide synthase	[27]
*pks*	*SMAC_03130*	Polyketide synthase (melanin biosynthesis)	[10]
*sdh*	*SMAC_02101*	Scytalone dehydratase (melanin biosynthesis)	[10]
*tih*	*SMAC_05650*	Trihydroxynaphtalene reductase (melanin biosynthesis)	[10, 22]
Transcription factors and mating-type genes			
*asm2*	*SMAC_09436*	GAL4-like zinc cluster transcription factor	[38]
*mcm1*	*SMAC_05219*	MADS-box transcription factor	[8]
*pro1*	*SMAC_00338*	C_6_ zinc finger transcription factor	[1]
*pro44*	*SMAC_03223*	GATA-type transcription factor	[22, 38]
*Smta-1*	*SMAC_05404*	MAT1–2-1, HMG domain transcription factor	[9]
*SmtA-2*	*SMAC_05402*	MAT1–1-2, PPF domain protein	[18]
*ste12*	*SMAC_06479*	Homeodomain/zinc finger transcription factor	[7]
Chromatin modifiers			
*asf1*	*SMAC_08608*	H3/H4 histone chaperone	[21, 38]
*cac2*	*SMAC_01629*	Putative subunit of CAF-1 (chromatin assembly factor 1)	[21, 38, 39]
*crc1*	*SMAC_02795*	CRC domain protein	[38, 39]
*rtt106*	*SMAC_03589*	Putative H3/H4 histone chaperone	[21, 38, 39]
*scm1*	*SMAC_04946*	SAS4 domain protein	[39]
*spt3*	*SMAC_01829*	Putative subunit of the SAGA complex	[39]
Pheromones and pheromone receptors			
*ppg1*	*SMAC_05970*	Peptide pheromone	[5]
*ppg2*	*SMAC_12967*	Lipopeptide pheromone	[6]
*pre1*	*SMAC_02283*	Pheromone receptor	[6]
*pre2*	*SMAC_08994*	Pheromone receptor	[6]
Signal transduction			
*gsa1*	*SMAC_05328*	G protein α-subunit	[13]
*gsa2*	*SMAC_06605*	G protein α-subunit	[13]
*gsa3*	*SMAC_07195*	G protein α-subunit	[13]
*sac1*	*SMAC_01638*	Adenylate cyclase	[13]
Subunits of the STRIPAK complex			
*pp2Ac1*	*SMAC_04678*	Catalytic subunit of protein phosphatase 2A	[33]
*pro11*	*SMAC_08794*	Striatin	[3]
*pro22*	*SMAC_02580*	STRIP	[17, 20]
*pro45*	*SMAC_01224*	SLMAP	[31]
*SCI1*	*SMAC_05559*	STRIPAK complex interactor 1	[37]
*Smgpi1*	*SMAC_12074*	GPI-anchored protein	[29]
*Smkin3*	*SMAC_04490*	Germinal center kinase	[30, 36]
*Smkin24*	*SMAC_01456*	Germinal center kinase	[30]
*Smmob3*	*SMAC_00877*	Phocein	[19]
MAPK signaling			
*mak1*	*SMAC_05504*	MAPK of CWI pathway	[28]
*mek1*	*SMAC_02183*	MAPK kinase (MAPKK) of CWI pathway	[28]
*mik1*	*SMAC_03673*	MAPK kinase kinase (MAPKKK) of CWI pathway	[28]
*pro40*	*SMAC_04815*	Scaffold protein for CWI pathway	[11, 28]
NOX complex			
*nor1*	*SMAC_02124*	NOX regulator	[25]
*nox1*	*SMAC_05007*	NADPH oxidase	[25]
*nox2*	*SMAC_08741*	NADPH oxidase	[25]
*pro41*	*SMAC_04848*	ER membrane protein, NoxD homolog	[12]
Genes without known domains or known molecular functions			
*spd4*	*SMAC_01964*	–	[35]

*CWI*, cell wall integrity pathway; *ER*, endoplasmic reticulum; *GPI*, glycosylphosphatidylinositol; *MAPK*, mitogen-activated protein kinase; *NOX*, NADPH oxidase; *SAGA*, Spt-Ada-Gcn5 histone acetyltransferase; *SLMAP*, sarcolemmal membrane-associated protein; *SNARE*, soluble N-ethylmaleimide-sensitive-factor attachment protein receptor; *STRIPAK*, striatin-interacting phosphatase and kinase; *STRIP*, striatin-interacting protein

About 15 years ago, *S. macrospora* entered the “omics” age, first in the form of transcriptomics by cross-species microarray hybridization. This technique made use of the close phylogenetic relationship between *S. macrospora* and *Neurospora crassa* (Nowrousian et al. 2004), which meant that available microarrays with *N. crassa* probes (Lewis et al. 2002; Nowrousian et al. 2003) could be hybridized with targets derived from *S. macrospora* (Nowrousian et al. 2005). Cross-species hybridization was used to characterize expression profiles of the wild type and several developmental mutants (Nowrousian et al. 2005; Pöggeler et al. 2006; Nowrousian et al. 2007; Klix et al. 2010).

Ten years ago, *S. macrospora* was the second eukaryote (the first was the giant panda (Li et al. 2010)), and the first fungus, to have its genome sequenced solely by next-generation sequencing techniques (Nowrousian et al. 2010). By now, two updated versions of the genome sequence have been published with improved annotation, the first based on RNA-seq, and the second based on proteogenomics (Teichert et al. 2012; Blank-Landeshammer et al. 2019). The availability of a genome sequence enabled the use of many other sequencing-based techniques for *S. macrospora*. One was the identification of defective genes in mutant strains by genome sequencing instead of the more laborious complementation by transformation. Mutant sequencing was done as bulk segregant analysis, a powerful approach that is possible only in model organisms where classical genetics can be combined with molecular and bioinformatics methods. Bulk segregant analysis was used to identify the developmental genes *mik1*, *nox1*, *pro44*, *spd4*, and *tih* (Nowrousian et al. 2012; Dirschnabel et al. 2014; Teichert et al. 2014b; Teichert et al. 2017b). The availability of a genome sequence also facilitated the use of RNA-seq for transcriptomics studies. Laser microdissection in combination with RNA-seq was used to analyze gene expression patterns in developing fruiting bodies in *S. macrospora* alone or in comparative transcriptomics studies where gene expression during development was compared between *S. macrospora* and other fungal species to identify evolutionary conserved expression patterns (Teichert et al. 2012; Traeger et al. 2013; Dirschnabel et al. 2014; Schumacher et al. 2018; Lütkenhaus et al. 2019).

Differentially expressed genes identified through transcriptomics of mutants or comparative transcriptomics of different species turned out to be good candidates for further functional analyses to identify novel developmental genes by reverse genetics. The generation of deletion mutants by homologous recombination for such candidate genes was facilitated by the availability of a Δku70 strain, which has a lower frequency of ectopic integration, leading to a higher proportion of targeted deletions in the resulting transformants (Pöggeler and Kück 2006). Deletion of differentially expressed candidate genes led to the identification of developmental genes *asf1*, *asm2*, *crc1*, *scm1*, *sec22*, *SMAC_06770*, *SmJLB1*, and *spt3* (Gesing et al. 2012; Voigt et al. 2013; Traeger and Nowrousian 2015; Schumacher et al. 2018; Lütkenhaus et al. 2019).

In addition to “omics”-based methods, fluorescence microscopy was used extensively to study fruiting body development in *S. macrospora*. For this purpose, the use of the green fluorescent protein (GFP) and fluorescent proteins for other wavelengths was established in this fungus, and also the use of superresolution structured illumination microscopy (SIM) to identify the subcellular localization of developmental proteins (Pöggeler et al. 2003; Rech et al. 2007; Nordzieke et al. 2015).

By 2020, 58 developmental genes have been identified and characterized in *S. macrospora*, and a better picture of the molecular processes involved in fruiting body formation is starting to emerge (Table 1, Fig. 2). Several previous reviews describe earlier studies using *S. macrospora* as a model organism (Kück et al. 2009; Engh et al. 2010; Teichert et al. 2014a), and therefore, the next sections of this review will focus on more recent results.

## Analysis of developmental mutants reveals the role of multi-protein complexes in fruiting body development

**Fig. 3:**
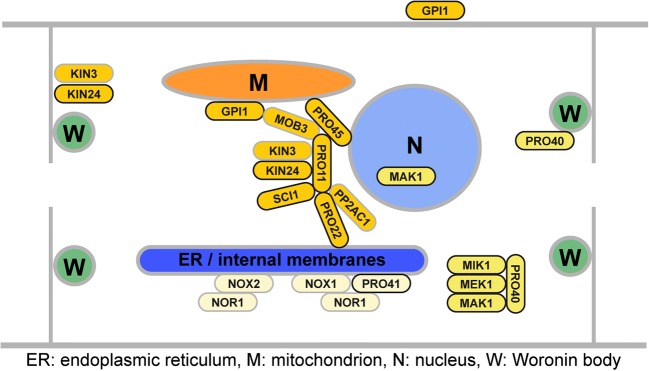
Components of the striatin-interacting phosphatase and kinase (STRIPAK) complex, the cell wall integrity (CWI) mitogen-activated kinase (MAPK) cascade, and two NADPH oxidase (NOX) complexes in *S. macrospora*. For coloring of proteins see key to Fig. 2

One of the major advantages when working with *S. macrospora* is the availability of two large mutant libraries containing developmental mutants blocked at different stages of fruiting body formation. These mutants were generated by classical mutagenesis, using X-ray, UV light, and ethyl methanesulfonate (EMS) (Esser and Straub 1958; Kück et al. 2009). By characterizing these mutants, we found base substitutions or deletions in protein-coding genes that are essential for fruiting body formation. Research with *S. macrospora* has mostly focused on “pro” mutants that are blocked after the formation of protoperithecia (Fig. 2). Starting from single PRO proteins, further research revealed links between different PRO proteins and their interaction partners, leading to the identification of several multi-protein complexes regulating sexual development. This section will focus on the striatin-interacting phosphatase and kinase (STRIPAK) complex, the cell wall integrity (CWI) mitogen-activated kinase (MAPK) cascade, and two NADPH oxidase (NOX) complexes and their role in sexual development of *S. macrospora* (Fig. 3).

The STRIPAK complex is a highly conserved eukaryotic protein complex assembled around the trimeric protein phosphatase 2A (PP2A) (Goudreault et al. 2009). This phosphatase consists of three subunits, the scaffolding subunit PP2AA, a catalytic subunit, and a regulatory subunit. Different regulatory subunits target the phosphatase to specific proteins. The first STRIPAK subunit characterized in fungi was PRO11, homologous to human striatins that serve as regulatory PP2A subunits within STRIPAK (Pöggeler and Kück 2004; Bloemendal et al. 2012). Complementation of the pro11 mutant with cosmids from an indexed library (Pöggeler et al. 1997) led to the identification of a premature stop codon in the mutant pro11 ORF (Pöggeler and Kück 2004). Similarly, a premature stop codon in mutant pro22 was detected, and later studies identified an interaction between PRO11 and PRO22 (Bloemendal et al. 2010; Bloemendal et al. 2012). PRO22 is homologous to striatin-interacting proteins and also belongs to the STRIPAK core, similar to one of two PP2A catalytic subunits, PP2Ac1 (Beier et al. 2016). Additional associated subunits include the SLMAP homolog PRO45, the phocein homolog MOB3, germinal center kinases Smkin3 and Smkin24, the small coiled-coil protein SCI1, and the GPI-anchored protein Smgpi1 (Bernhards and Pöggeler 2011; Frey et al. 2015a; Frey et al. 2015b; Nordzieke et al. 2015; Radchenko et al. 2018; Reschka et al. 2018) (see Table 1). A common phenotype of core STRIPAK mutants in *S. macrospora* is their lack of fruiting bodies, development arrests after the formation of protoperithecia (Kück et al. 2016). This phenotype is shared with Δmob3, Δpro45, and Δsci1 (Bernhards and Pöggeler 2011; Nordzieke et al. 2015; Reschka et al. 2018). Besides a general role in fruiting body formation, distinct STRIPAK subunits have additional functions, e.g., PRO22, PP2Ac1, and SmKIN3 in ascogonial septum formation (Bloemendal et al. 2010; Beier et al. 2016; Radchenko et al. 2018).

The cell wall integrity pathway was characterized when genome sequencing of mutant pro30 revealed that a defect in the MAPK kinase (MAPKK) gene *mek1* caused the sterile phenotype (Teichert et al. 2014b). Protein-protein interaction studies showed that the pathway consists of MAPK kinase kinase (MAPKKK) MIK1, MAPKK MEK1, and MAPK MAK1 (see Table 1). Further upstream components include protein kinase C (PKC1) and the small G protein RHO1 (Teichert et al. 2014b). Furthermore, it was shown that MAPKK MEK1 interacts with PRO40, a protein that had previously been described as being essential for fruiting body formation and hyphal fusion (Engh et al. 2007b, Rech et al. 2007). PRO40 interacts with MEK1, MIK1, and PKC1. MIK1 and PKC1 do not interact in a yeast two-hybrid assay; thus, PRO40 mediates the interaction of the MAPK module with the upstream kinase (Teichert et al. 2014b). In vivo phosphorylation assays showed that phosphorylation of the MAPK MAK1 is strongly reduced in Δpro40 during development and stress response. Therefore, PRO40 acts as a scaffold protein for the CWI MAPK pathway during these processes. However, yet unknown scaffold proteins are required for CWI pathway functions in cell wall integrity.

Studies of the *S. macrospora* NOX complexes include the analysis of the two mutants pro32, showing a defect in the *nox1* gene, and pro41, having a defect in the *pro41* gene that is homologous to *noxD* from *Botrytis cinerea* and *Podospora anserina* (Nowrousian et al. 2007; Dirschnabel et al. 2014). NOXD is a membrane protein interacting with NOX1 homologs in fungi and in higher eukaryotes (Lacaze et al. 2015; Siegmund et al. 2015). NOXs are important for the generation of reactive oxygen species (ROS) by oxidation of NADPH (Lambeth 2004). Importantly, it has been hypothesized that ROS do not only act in a damaging way, but that they also act as signaling molecules that drive developmental processes (Scott and Eaton 2008; Aguirre and Lambeth 2010; Heller and Tudzynski 2011). Two NOX complexes exist in *S. macrospora*, the NOX1 complex containing NOX1 and most probably PRO41 as well as the NOX2 complex containing NOX2. Both complexes further contain the NOX regulator NOR1 (Dirschnabel et al. 2014). The NOX1 complex controls the maturation of protoperithecia to perithecia, vegetative hyphal fusion, and vegetative growth as well as oxidative stress response. The NOX2 complex regulates the germination of ascospores. Interestingly, *nox2* and *nor1* deletion mutants germinate only in a melanin-deficient background. This phenomenon may be caused by quenching of residual reactive oxygen species by melanin in the ascospore cell wall. In a brown-spored fus mutant defective of melanin production, the residual ROS may be sufficient to trigger germination (Dirschnabel et al. 2014).

Taken together, several developmental mutants have aided in the identification of multi-protein complexes that control fruiting body formation. These results highlight the advantages of forward genetic screens for the characterization of distinct developmental processes.

## Autophagy: the role of cellular recycling in fruiting body development

**Fig. 4:**
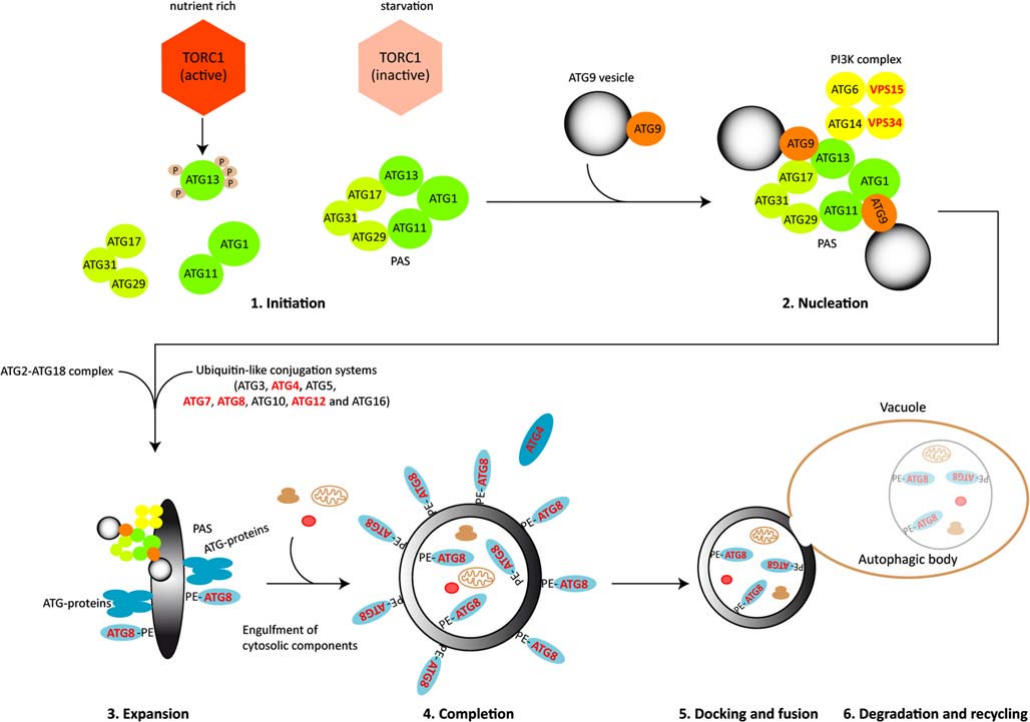
Major stages in non-selective autophagy in *S. cerevisiae*. The process of autophagy can be divided into six steps: (1) Under nutrient-rich conditions, the active TOR kinase phosphorylates ATG13. Hyperphosphorylation of ATG13 prevents an interaction with the kinase ATG1 and the scaffold protein ATG17. After inactivation of TOR during starvation or rapamycin treatment, the hypophosphorylated ATG13 forms a complex with the ATG1 kinase and the tripartite ATG17-ATG31-ATG29 complex as well as the adaptor protein ATG11 at the phagophore assembly site (PAS). (2) Nucleation involves recruitment of ATG9 vesicles and the phosphoinositide-3-kinase (PI3K) complex composed of ATG6, ATG14, and the vacuolar sorting proteins VPS34 and VPS15. (3) Expansion of the PAS to a phagophore requires the recruitment of the ATG2-ATG18 complex and the ubiquitin-like (Ubl) conjugation systems (consisting of ATG3, ATG4 ATG5, ATG7, ATG8, ATG10, ATG12, and ATG16). The Ubl conjugation systems are needed for the conjugation of ATG8 and phosphatidylethanolamine (PE) which allows the conjugate ATG8-PE to be anchored at the convex and concave site of the phagophore. (4) Extension of the phagophore membrane results in the engulfment of cytosolic compounds and completion of the double-membraned autophagosome. (5) The outer membrane of mature autophagosomes fuses with the vacuolar membrane and releases the single-membraned autophagic body into the lumen of the vacuole. (6) Hydolases degrade the autophagic body and its content, and permeases recycle molecular building blocks back into the cytoplasm. Proteins analyzed in *S. macrospora* are indicated in red letters

All eukaryotes, including fungi, have conserved mechanisms for recycling cytosolic material in times of stress or during cellular differentiation processes. Recent findings have revealed that autophagy (literally, self-eating) plays a key role in fungal development and pathogenicity (Bartoszewska and Kiel 2011; Voigt and Pöggeler 2013b; Pöggeler et al. 2018; Zhu et al. 2019). During autophagy, a double lipid bilayer surrounds parts of the cytoplasm including organelles, expands, closes, and forms a double-membraned vesicle, called the autophagosome (Fig. 4). These autophagosomes are delivered to the vacuole for degradation. The outer membrane of the autophagosome fuses with the vacuolar membrane and releases the single-membraned autophagic body into the lumen of the vacuole. In the vacuole, the autophagic body and its content are hydrolyzed by vacuolar enzymes, and metabolites are transported back into the cytoplasm via vacuolar permeases for reuse (Wen and Klionsky 2016).

Autophagy relies on autophagy-related genes (*atg*) genes. The process requires in yeast 19 core ATG proteins that regulate initiation, nucleation and expansion of the phagophore membrane, completion of the autophagosome, docking and fusion with the vacuolar membrane, degradation, and recycling (Fig. 4) (Ohsumi 2014). Most of these core *atg* genes are conserved among eukaryotes including filamentous fungi (Bartoszewska and Kiel 2011; Voigt and Pöggeler 2013b). The random engulfment of cytoplasm by autophagosomes is called non-selective autophagy. In addition, selective autophagy leads to the specific, vacuolar degradation of superfluous or damaged organelles like ribosomes, peroxisomes, mitochondria, nuclei, and the ER, by ribophagy, pexophagy, mitophagy, nucleophagy, and ER-phagy, respectively (Farré and Subramani 2016).

Mining of the published *S. macrospora* genome sequence (Nowrousian et al. 2010) revealed the coding capacity for 17 core *atg* genes. However, the *S. macrospora* genome does not encode clear orthologs of *Saccharomyces cerevisiae* ATG proteins required for selective autophagy (Voigt and Pöggeler 2013b; Teichert et al. 2014a).

To determine whether non-selective autophagy is necessary for vegetative growth and fruiting body development in *S. macrospora*, we characterized the function of core autophagic proteins of the Ubl conjugation systems. Budding yeast has two ubiquitin-like-conjugation systems, which are involved in autophagosome formation and expansion. The ubiquitin-like protein ATG12 is covalently attached to ATG5 by the action of the E1-like enzyme ATG7 and the E2-like enzyme ATG10. In the second conjugation system, the ubiquitin-like protein ATG8 is C-terminally processed by the cysteine protease ATG4 and then activated by ATG7 and transferred to the E2-like enzyme ATG3. Finally, a conjugate of ATG8 and phosphatidylethanolamine (PE) is formed by the E3-like complex ATG12/ATG5/ATG16. The ATG8-PE conjugate is a structural component of the outer and inner autophagosomal membrane (Fig. 4) (Ohsumi 2001). In addition to processing the ATG8 precursor, the ATG4 protease acts as a deconjugating enzyme and facilitates the recycling of ATG8 from the outer membrane of the autophagosome (Kirisako et al. 2000).

In *S. macrospora*, we were not able to generate a homokaryotic ΔSmatg7 mutant, suggesting that E1-like enzyme SmATG7 is required for viability (Nolting et al. 2009). However, we succeeded to delete *Smatg4*, *Smatg8*, and *Smatg12*. All three homokaryotic deletion mutants are impaired in vegetative growth and stop fruiting body development at the stage of protoperithecia formation. In this regard, core autophagy mutants resemble pro mutants of *S. macrospora*; though in contrast to many pro mutants, they are not impaired in hyphal fusion (Voigt and Pöggeler 2013a; Werner et al. 2016). For SmATG4, we demonstrated that the protease is capable of processing SmATG8. Localization studies revealed that SmATG8 localizes to autophagosomes, SmATG12 to phagophores, and SmATG4 is distributed throughout the cytoplasm. We could also show that *Smatg8* and *Smatg4* are required not only for non-selective macroautophagy, but for selective pexophagy as well (Voigt and Pöggeler 2013a). *Smatg4* and *Smatg8* genes are transcriptionally repressed by the bZIP transcription factor SmJLB1 (Voigt et al. 2013). Moreover, we showed that *vps34* and *vps15*, encoding components of the PI3K complex and the vacuolar protein sorting complex, are required for viability of *S. macrospora* (Voigt et al. 2014). These results suggest that in *S. macrospora*, autophagy is an essential and constitutively active process required to maintain high energy levels for filamentous growth and multicellular development.

To examine to which extent selective autophagy contributes to fungal fruiting body development, we applied a GFP-Trap analysis with EGFP-SmATG8 to identify SmATG8-interacting proteins. Among proteins expected to interact with ATG8, such as ATG3 and ATG7, the mammalian homolog of the mammalian cargo receptor NBR1, SmNBR1, was identified as an interaction partner of SmATG8 (Werner et al. 2019). In mammals, NBR1 is a ubiquitously expressed conserved protein that is associated with signaling pathways and bone metabolism (Yin et al. 2019). It was also described as a binding partner of Atg8 family proteins where it acts as a receptor that mediates the docking of ubiquitinated substrates to the autophagosomal membrane protein LC3/ATG8 (Kirkin et al. 2009; Lamark et al. 2009). In addition, in mammals, NBR1 is involved in pexophagy and aggrephagy, as well as putatively in mitophagy (Deosaran et al. 2013; Rogov et al. 2014). The mammalian NBR1 has an N-terminal PB1 (Phox and Bem1) domain followed by a ZZ-type zinc finger domain, coiled-coil domains, an Atg8-interacting motif LIR (LC3 interacting region), and a C-terminal UBA (ubiquitin-associated) domain. Furthermore, a functionally uncharacterized NBR1 domain with four conserved tryptophan residues is a hallmark of all NBR1-like proteins (Kraft et al. 2010; Svenning et al. 2011). In plants, NBR1 plays important roles in stress response and aggrephagy (Svenning et al. 2011; Zhou et al. 2013). With the exception of Saccharomycetes, putative homologs of NBR1 have been identified throughout the eukaryotic kingdom (Svenning et al. 2011). Filamentous ascomycetes and basidiomycetes have retained a homolog of NBR1 in which the C-terminal UBA domain is absent (Kraft et al. 2010). Thus, the *S. macrospora* protein SmNBR1 lacks a UBA domain but displays all the characteristic domains of NBR1, and contains a repetition of the ZZ domain. In vivo co-localization, bimolecular fluorescence complementation (BiFC) experiments, and co-immunoprecipitation (CoIP) verified interaction of SmATG8 and SmNBR1. SmNBR1 accumulates in cytosolic “aggregates” and in vacuoles of young and old hyphae and co-localizes with SmATG8. A yeast two-hybrid analysis revealed that the LIR motif of SmNBR1 is required for the interaction with SmATG8. In a ΔSmatg8 mutant, SmNBR1 localizes to cytoplasmic aggregates or is homogenously distributed in the cytoplasm, but it was not detected in vacuoles. These findings indicate that in the absence of SmATG8 and functional autophagy, SmNBR1 cannot be transported into vacuoles.

Deletion of *Smnbr1* leads to impaired vegetative growth under starvation conditions. Moreover, fruiting body development was significantly delayed in the ΔSmnbr1 mutant. Ascogonia and protoperithecia formed only after a prolonged time and in reduced numbers. Although transition to perithecia was observed in the ΔSmnbr1 mutant, fewer perithecia were formed in comparison with the wild type. Fruiting bodies formed in ΔSmnbr1 contained drastically reduced numbers of mature, black ascospores. Thus, *Smnbr1* is required for proper vegetative growth, fruiting body, and ascospore differentiation. Similar to observations made in mammals (Deosaran et al. 2013), we could show that in contrast to the wild type, labeled peroxisomes were not delivered to vacuoles in the ΔSmnbr1 mutant. This suggests that SmNBR1 is a pexophagy receptor (Werner et al. 2019). The human NBR1 homolog partially rescues the phenotypic defects of the *S. macropora* ΔSmnbr1 deletion mutant, indicating a functional conservation of NBR1 homologs across eukaryotic kingdoms (Werner et al. 2019).

Only a few reports describe the role of selective autophagy in fungi other than in *S. cerevisiae*. Our results support the hypothesis that selective autophagy plays an important role in differentiation of filamentous fungi and that the cargo receptor NBR1 is a central component of selective autophagy. Analysis of selective autophagy in *S. macrospora* will help to understand how selective recycling of proteins, protein complexes, and organelles via autophagy and complex differentiation processes are mechanistically linked and regulated.

## Transcription factors and the role of chromatin in fruiting body development

One of the first identified developmental genes in *S. macrospora* was the transcription factor gene *pro1* (Masloff et al. 1999). Although PRO1 was hypothesized early on to be involved in regulating the expression of downstream genes involved in fruiting body development (Masloff et al. 1999; Masloff et al. 2002), direct target genes of PRO1 were identified only years later by ChIP-seq (Steffens et al. 2016). These analyses confirmed that indeed PRO1 is involved in regulating the expression of a number of genes with a role in fruiting body morphogenesis.

Transcriptomics analysis of young fruiting bodies of the wild type and the pro1 mutant identified more than 400 genes that are differentially regulated during development and are dependent on *pro1* for correct expression, although this control might be indirect (Teichert et al. 2012). One of these genes encodes the developmental transcription factor PRO44, which was subsequently shown to be strongly expressed in cells of the outer layers of the developing fruiting body (Schumacher et al. 2018). Transcriptomics analyses of young fruiting bodies of a Δpro44 mutant identified another transcription factor gene, *asm2*, which is downregulated in Δpro44 fruiting body precursors. Deletion of *asm2* leads to problems late in fruiting body development, namely with ascospore maturation and discharge (Schumacher et al. 2018). Thus, the three transcription factor genes *pro1*, *pro44*, and *asm2* are part of a genetic network regulating the progression of fruiting body development.

Another finding of genome-wide expression analyses in young fruiting bodies was that gene expression patterns in young fruiting bodies differ greatly from those of vegetative mycelia (Teichert et al. 2012; Schumacher et al. 2018). This finding is not restricted to *S. macrospora*, but can be observed also in comparative transcriptomics studies with other, distantly related ascomycetes, allowing for the identification of evolutionary conserved expression patterns during development (Lütkenhaus et al. 2019). Since ascomycete fruiting bodies contain many cell types that do not occur in vegetative mycelia (Bistis et al. 2003; Lord and Read 2011), it is thought that the massive changes in the transcriptome might reflect the requirement for major changes in metabolic activity and their regulation during the formation of the fruiting body. Transcription factors will be involved in mediating these changes; however, results in recent years indicate that other factors that influence the structure and function of chromatin also play a role in fruiting body development.

In *S. macrospora*, the first chromatin modifier to be identified as a developmental gene was *asf1*, which encodes a histone H3/H4 chaperone (Gesing et al. 2012). Histone chaperones comprise a diverse group of proteins that handle non-nucleosomal histones in vivo, and in vitro can mediate the assembly of nucleosomes from isolated histones and DNA (Hammond et al. 2017). A Δasf1 mutant has a block during early stages of fruiting body development, and transcriptomics analyses of the mutant showed that *asf1* acts as a suppressor of weakly expressed genes during development (Gesing et al. 2012; Schumacher et al. 2018). How ASF1 might mediate gene expression changes is not clear yet. Genome-wide analyses of nucleosome positioning as well as cytosine methylation in the Δasf1 mutant compared with the wild type failed to identify changes that would explain the expression changes specifically in weakly expressed genes (Schumacher et al. 2018); therefore, additional analyses, for example of histone modifications, will be needed to address this question.

Another chromatin modifier recently identified to be required for fruiting body formation in *S. macrospora* is *spt3* (Lütkenhaus et al. 2019). *spt3* encodes a homolog to the SPT3 subunit of the SAGA complex, which is a conserved eukaryotic transcriptional co-activator complex with multiple functions in histone modification and interactions with other transcriptional activators (Spedale et al. 2012; Helmlinger and Tora 2017). A Δspt3 mutant shows reduced vegetative growth and an early block during fruiting body development, similar to a previously characterized *spt3* mutant of *Fusarium graminearum* (Gao et al. 2014; Lütkenhaus et al. 2019).

Several other putative chromatin modifier genes were analyzed for their role in sexual development in *S. macrospora*, but none of them showed such a drastic phenotype as observed for *asf1* and *spt3*. Single mutants of the genes for the histone H3/H4 chaperones *cac2* and *rtt106*, as well as the putative chromatin modifiers *crc1* and *scm1*, were fertile, and even all combinations of double mutants turned out to be fertile (Gesing et al. 2012; Schumacher et al. 2018; Lütkenhaus et al. 2019). However, all possible combinations of triple mutants showed different degrees of reduction in fertility, and the quadruple mutant Δcac2/Δcrc1/Δrtt106/Δscm1 turned out to be completely sterile with a block during early stages of fruiting body development (Lütkenhaus et al. 2019). Addressing the question if the corresponding proteins have redundant roles in the same pathway or are essential components of different regulatory pathways with redundant functions will be part of future studies. Another important question for future analyses is how the activity of chromatin modifiers is integrated with the activity of specific transcription factors like PRO1, PRO44, or ASM2.

## RNA editing: a new paradigm in fruiting body development of filamentous ascomycetes

RNA editing comprises nucleotide substitutions or short insertions/deletions within transcripts that could alternatively be directly encoded at the DNA level (Knoop 2011). Editing occurs in diverse RNA species across kingdoms. The existence of RNA editing of nuclear-encoded protein-coding transcripts was only recently described in fungi (Liu et al. 2016; Liu et al. 2017; Teichert et al. 2017a). By a yet unknown catalytic activity, adenosin (A) is desaminated to inosin (I). Since I pairs with cytidine, the structure of edited RNAs can differ from the native, genome-encoded structure. Furthermore, the ribosome interprets I as guanosin (G), which may lead to amino acid exchanges in the encoded proteins.

First evidence that *S. macrospora* transcripts undergo A-to-I RNA editing came from RNA-seq data generated from wild type and mutant protoperithecia (Teichert et al. 2012; Dirschnabel et al. 2014; Teichert et al. 2017a). In wild type protoperithecia, but not in wild type vegetative mycelial samples or in mutant protoperithecia, the level of A-to-G exchanges indicative of A-to-I editing increased significantly (Teichert et al. 2017a). This is in accordance with data from other filamentous ascomycetes, where editing levels increase with the progression of fruiting body formation (Liu et al. 2016; Liu et al. 2017; Teichert et al. 2017a).

Interestingly, editing sites in fungi occur mostly in coding regions, which is in contrast to metazoan species, where editing sites reside mostly in non-coding regions (Teichert 2018; Bian et al. 2019). Thus, fungal RNA editing mostly leads to coding changes, which in turn may lead to single amino acid variations (SAAVs). Indeed, SAAVs were identified in protein extracts from sexually developed cultures by proteogenomics analysis (Blank-Landeshammer et al. 2019). Quantitative mass spectrometry with synthetic peptides showed that the level of modified peptides increased with progression of fruiting body formation. This is in accordance with transcriptomics data and strengthens the hypothesis that editing may be required for late stages of fruiting body formation and the generation of ascospores.

Another feature of fungal editing is the targeting of stop codons. Either premature stop codons within wrongly annotated open reading frames are “corrected”, i.e., during fruiting body formation, a functional protein is formed, or the annotated translational stop is targeted, leading to C-terminal elongation of proteins (Liu et al. 2016; Liu et al. 2017; Teichert et al. 2017a). Like with SAAVs, peptides from C-terminally elongated proteins can be detected in protein extracts from cultures generating fruiting bodies (Blank-Landeshammer et al. 2019). In silico analysis predicts new linear motifs, localization signals, or even protein domains in these elongations. For example, the blue light sensor and transcription factor White Collar 1 gains a histone deacetylase domain by RNA editing of its transcripts at late stages of fruiting body formation (Blank-Landeshammer et al. 2019). Why proteins need these new functions during development, if A-to-I RNA editing is a prerequisite for ascospore formation, how editing is catalyzed in fungi, and how it evolved are open questions that have to be answered by future research.

## Conclusions

The year 2020 marks the 25th anniversary of *S. macrospora* entering the world of molecular biology as a model organism for fungal development. More than 50 developmental genes have been characterized during that time, and a better picture of the molecular processes involved in fruiting body development is starting to emerge. Future challenges include the open question of how the different regulatory and metabolic processes needed to build a fruiting body are orchestrated in time and space. However, with its complement of classical genetics, molecular, and “omics” tools and resources available, *S. macrospora* is an excellent model organism to tackle these exciting questions.

## References

[CR1] Aguirre J, Lambeth JD (2010). Nox enzymes from fungus to fly to fish and what they tell us about Nox function in mammals. Free Radic Biol Med.

[CR2] Bartoszewska M, Kiel JA (2011). The role of macroautophagy in development of filamentous fungi. Antioxid Redox Signal.

[CR3] Beier A, Teichert I, Krisp C, Wolters DA, Kück U (2016). Catalytic subunit 1 of protein phosphatase 2A is a subunit of the STRIPAK complex and governs fungal sexual development. mBio.

[CR4] Bernhards Y, Pöggeler S (2011). The phocein homologue SmMOB3 is essential for vegetative cell fusion and sexual development in the filamentous ascomycete *Sordaria macrospora*. Curr Genet.

[CR5] Bian Z, Ni Y, Xu JR, Liu H (2019). A-to-I mRNA editing in fungi: occurrence, function, and evolution. Cell Mol Life Sci.

[CR6] Bistis GN, Perkins DD, Read ND (2003). Different cell types in *Neurospora crassa*. Fungal Genet Newsl.

[CR7] Blank-Landeshammer B, Teichert I, Märker R, Nowrousian M, Kück U, Sickmann A (2019). Combination of proteogenomics with peptide *de novo* sequencing identifies new genes and hidden posttranscriptional modifications. mBio.

[CR8] Bloemendal S, Lord KM, Rech C, Hoff B, Engh I, Read ND, Kück U (2010). A mutant defective in sexual development produces aseptate ascogonia. Eukaryot Cell.

[CR9] Bloemendal S, Bernhards Y, Bartho K, Dettmann A, Voigt O, Teichert I, Seiler S, Wolters DA, Pöggeler S, Kück U (2012). A homolog of the human STRIPAK complex controls sexual development in fungi. Mol Microbiol.

[CR10] Deosaran E, Larsen KB, Hua R, Sargent G, Wang Y, Kim S, Lamark T, Jauregui M, Law K, Lippincott-Schwartz J, Brech A, Johansen T, Kim PK (2013). NBR1 acts as an autophagy receptor for peroxisomes. J Cell Sci.

[CR11] Dirschnabel DE, Nowrousian M, Cano-Domínguez N, Aguirre J, Teichert I, Kück U (2014). New insights into the roles of NADPH oxidases in sexual development and ascospore germination in *Sordaria macrospora*. Genetics.

[CR12] Elleuche S, Pöggeler S (2009). β-carbonic anhydrases play a role in fruiting body development and ascospore germination in the filamentous fungus *Sordaria macrospora*. PLOS One.

[CR13] Engh I, Nowrousian M, Kück U (2007). Regulation of melanin biosynthesis via the dihydroxynaphtalene pathway is dependent on sexual development in the ascomycete *Sordaria macrospora*. FEMS Microbiol Lett.

[CR14] Engh I, Würtz C, Witzel-Schlömp K, Zhang HY, Hoff B, Nowrousian M, Rottensteiner H, Kück U (2007). The WW domain protein PRO40 is required for fungal fertility and associates with Woronin bodies. Eukaryot Cell.

[CR15] Engh I, Nowrousian M, Kück U (2010) *Sordaria macrospora*, a model organism to study fungal cellular development. Eur J Cell Biol 89:864–872 10.1016/j.ejcb.2010.07.002 20739093

[CR16] Esser K, Straub J (1956). Fertility in the heterokaryon from two sterile mutants of *Sordaria macrospora* Auersw. Z Indukt Abstamm Vererbungsl.

[CR17] Esser K, Straub J (1958). Genetische Untersuchungen an *Sordaria macrospora* Auersw., Kompensation und Induktion bei genbedingten Entwicklungsdefekten. Z Vererbungsl.

[CR18] Farré JC, Subramani S (2016). Mechanistic insights into selective autophagy pathways: lessons from yeast. Nat Rev Mol Cell Biol.

[CR19] Frey S, Lahmann Y, Hartmann T, Seiler S, Pöggeler S (2015). Deletion of *Smgpi1* encoding a GPI-anchored protein suppresses sterility of the STRIPAK mutant ΔSmmob3 in the filamentous ascomycete *Sordaria macrospora*. Mol Microbiol.

[CR20] Frey S, Reschka EJ, Pöggeler S (2015). Germinal center kinases SmKIN3 and SmKIN24 are associated with the *Sordaria macrospora* striatin-interacting phosphatase and kinase (STRIPAK) complex. PLoS One.

[CR21] Gao T, Zheng Z, Hou Y, Zhou M (2014). Transcription factors spt3 and spt8 are associated with conidiation, mycelium growth, and pathogenicity in *Fusarium graminearum*. FEMS Microbiol Lett.

[CR22] Gesing S, Schindler D, Fränzel B, Wolters D, Nowrousian M (2012). The histone chaperone ASF1 is essential for sexual development in the filamentous fungus *Sordaria macrospora*. Mol Microbiol.

[CR23] Goudreault M, D’Ambrosio LM, Kean MJ, Mullin MJ, Larsen BG, Sanchez A, Chaudhry S, Chen GI, Sicheri F, Nesvizhskii AI, Aebersold R, Raught B, Gingras AC (2009). A PP2A phosphatase high density interaction network identifies a novel striatin-interacting phosphatase and kinase complex linked to the cerebral cavernous malformation 3 (CCM3) protein. Mol Cell Proteomics.

[CR24] Hammond CM, Strømme CB, Huang H, Patel DJ, Groth A (2017). Histone chaperone networks shaping chromatin function. Nat Rev Mol Cell Biol.

[CR25] Heller J, Tudzynski P (2011). Reactive oxygen species in phytopathogenic fungi: signaling, development, and disease. Annu Rev Phytopathol.

[CR26] Helmlinger D, Tora L (2017). Sharing the SAGA. Trends Biochem Sci.

[CR27] Kamerewerd J, Jansson M, Nowrousian M, Pöggeler S, Kück U (2008). Three alpha subunits of heterotrimeric G proteins and an adenylyl cyclase have distinct roles in fruiting body development in the homothallic fungus *Sordaria macrospora*. Genetics.

[CR28] Kirisako T, Ichimura Y, Okada H, Kabeya Y, Mizushima N, Yoshimori T, Ohsumi M, Takao T, Noda T, Ohsumi Y (2000). The reversible modification regulates the membrane-binding state of Apg8/Aut7 essential for autophagy and the cytoplasm to vacuole targeting pathway. J Cell Biol.

[CR29] Kirkin V, Lamark T, Sou YS, Bjørkøy G, Nunn JL, Bruun JA, Shvets E, McEwan DG, Clausen TH, Wild P, Bilusic I, Theurillat JP, Øvervatn A, Ishii T, Elazar Z, Komatsu M, Dikic I, Johansen T (2009). A role for NBR1 in autophagosomal degradation of ubiquitinated substrates. Mol Cell.

[CR30] Klix V, Nowrousian M, Ringelberg C, Loros JJ, Dunlap JC, Pöggeler S (2010). Functional characterization of *MAT1-1*-specific mating-type genes in the homothallic ascomycete *Sordaria macrospora* provides new insights into essential and non-essential sexual regulators. Eukaryot Cell.

[CR31] Knoop V (2011). When you can’t trust the DNA: RNA editing changes transcript sequences. Cell Mol Life Sci.

[CR32] Kraft C, Peter M, Hofmann K (2010). Selective autophagy: ubiquitin-mediated recognition and beyond. Nat Cell Biol.

[CR33] Kück U (2005). A *Sordaria macrospora* mutant lacking the *leu1* gene shows a developmental arrest during fruiting body formation. Mol Gen Genomics.

[CR34] Kück U, Pöggeler S, Nowrousian M, Nolting N, Engh I, Anke T, Weber D (2009). *Sordaria macrospora*, a model system for fungal development. The mycota XV, physiology and genetics.

[CR35] Kück U, Beier AM, Teichert I (2016). The composition and function of the striatin-interacting phosphatases and kinases (STRIPAK) complex in fungi. Fungal Genet Biol.

[CR36] Lacaze I, Lalucque H, Siegmund U, Silar P, Brun S (2015). Identification of NoxD/Pro41 as the homologue of the p22phox NADPH oxidase subunit in fungi. Mol Microbiol.

[CR37] Lamark T, Kirkin V, Dikic I, Johansen T (2009). NBR1 and p62 as cargo receptors for selective autophagy of ubiquitinated targets. Cell Cycle.

[CR38] Lambeth JD (2004). NOX enzymes and the biology of reactive oxygen. Nat Rev Immunol.

[CR39] Lehneck R, Elleuche S, Pöggeler S (2014). The filamentous ascomycete *Sordaria macrospora* can survive in ambient air without carbonic anhydrases. Mol Microbiol.

[CR40] Lewis ZA, Correa A, Schwerdtfeger C, Link KL, Xie X, Gomer RH, Thomas T, Ebbole DJ, Bell-Pedersen D (2002). Overexpression of white collar-1 (WC-1) activates circadian clock-associated genes, but is not sufficient to induce most light-regulated gene expression in *Neurospora crassa*. Mol Microbiol.

[CR41] Li R, Fan W, Tian G, Zhu H, He L, Cai J, Huang Q, Cai Q, Li B, Bai Y, Zhang Z, Zhang Y, Wang W, Li J, Wei F, Li H, Jian M, Li J, Zhang Z, Nielsen R, Li D, Gu W, Yang Z, Xuan Z, Ryder OA, Leung FC-C, Zhou Y, Cao J, Sun X, Fu Y, Fang X, Guo X, Wang B, Hou R, Shen F, Mu B, Ni P, Lin R, Qian W, Wang G, Yu C, Nie W, Wang J, Wu Z, Liang H, Min J, Wu Q, Cheng S, Ruan J, Wang M, Shi Z, Wen M, Liu B, Ren X, Zheng H, Dong D, Cook K, Shan G, Zhang H, Kosiol C, Xie X, Lu Z, Zheng H, Li Y, Steiner CC, Lam TT-Y, Lin S, Zhang Q, Li G, Tian J, Gong T, Liu H, Zhang D, Fang L, Ye C, Zhang J, Hu W, Xu A, Ren Y, Zhang G, Bruford MW, Li Q, Ma L, Guo Y, An N, Hu Y, Zheng Y, Shi Y, Li Z, Liu Q, Chen Y, Zhao J, Qu N, Zhao S, Tian F, Wang X, Wang H, Xu L, Liu X, Vinar T, Wang Y, Lam T-W, Yiu S-M, Liu S, Zhang H, Li D, Huang Y, Wang X, Yang G, Jiang Z, Wang J, Qin N, Li L, Li J, Bolund L, Kristiansen K, Wong GK-S, Olson M, Zhang X, Li S, Yang H, Wang J, Wang J (2010). The sequence and *de novo* assembly of the giant panda genome. Nature.

[CR42] Liu H, Wang Q, He Y, Chen L, Hao C, Jiang C, Li Y, Dai YC, Kang Z, Xu JR (2016). Genome-wide A-to-I RNA editing in fungi independent of ADAR enzymes. Genome Res.

[CR43] Liu H, Li Y, Chen DC, Qi Z, Wang Q, Wang J, Jiang C, Xu JR (2017). A-to-I RNA editing is developmentally regulated and generally adaptive for sexual reproduction in *Neurospora crassa*. Proc Natl Acad Sci U S A.

[CR44] Lord KM, Read ND (2011). Perithecium morphogenesis in *Sordaria macrospora*. Fungal Genet Biol.

[CR45] Lütkenhaus R, Traeger S, Breuer J, Carreté L, Kuo A, Lipzen A, Pangilinan J, Dilworth D, Sandor L, Pöggeler S, Gabaldón T, Barry K, Grigoriev IV, Nowrousian M (2019) Comparative genomics and transcriptomics to analyze fruiting body development in filamentous ascomycetes. Genetics 213:1545–1563. 10.1534/genetics.119.302749 10.1534/genetics.119.302749 PMC6893386 31604798

[CR46] Masloff S, Pöggeler S, Kück U (1999). The *pro1+* gene from *Sordaria macrospora* encodes a C_6_ zinc finger transcription factor required for fruiting body development. Genetics.

[CR47] Masloff S, Jacobsen S, Pöggeler S, Kück U (2002). Functional analysis of the C_6_ zinc finger gene *pro1* involved in fungal sexual development. Fungal Genet Biol.

[CR48] Mayrhofer S, Pöggeler S (2005). Functional characterization of an {alpha}-factor-like *Sordaria macrospora* peptide pheromone and analysis of its interaction with its cognate receptor in *Saccharomyces cerevisiae*. Eukaryot Cell.

[CR49] Mayrhofer S, Weber JM, Pöggeler S (2006). Pheromones and pheromone receptors are required for proper sexual development in the homothallic ascomycete *Sordaria macrospora*. Genetics.

[CR50] Nagy LG, Kovács GM, Krizsán K (2018). Complex multicellularity in fungi: evolutionary convergence, single origin, or both. Biol Rev.

[CR51] Nolting N, Pöggeler S (2006). A STE12 homologue of the homothallic ascomycete *Sordaria macrospora* interacts with the MADS box protein MCM1 and is required for ascosporogenesis. Mol Microbiol.

[CR52] Nolting N, Pöggeler S (2006). A MADS box protein interacts with a mating-type protein and is required for fruiting body development in the homothallic ascomycete *Sordaria macrospora*. Eukaryot Cell.

[CR53] Nolting N, Bernhards Y, Pöggeler S (2009). SmATG7 is required for viability in the homothallic ascomycete *Sordaria macrospora*. Fungal Genet Biol.

[CR54] Nordzieke S, Zobel T, Fränzel B, Wolters DA, Kück U, Teichert I (2015). A fungal SLMAP homolog plays a fundamental role in development and localizes to the nuclear envelope, ER, and mitochondria. Eukaryot Cell.

[CR55] Nowrousian M (2009). A novel polyketide biosynthesis gene cluster is involved in fruiting body morphogenesis in the filamentous fungi *Sordaria macrospora* and *Neurospora crassa*. Curr Genet.

[CR56] Nowrousian M, Masloff S, Pöggeler S, Kück U (1999). Cell differentiation during sexual development of the fungus *Sordaria macrospora* requires ATP citrate lyase activity. Mol Cell Biol.

[CR57] Nowrousian M, Duffield GE, Loros JJ, Dunlap JC (2003). The *frequency* gene is required for temperature-dependent regulation of many clock-controlled genes in *Neurospora crassa*. Genetics.

[CR58] Nowrousian M, Würtz C, Pöggeler S, Kück U (2004). Comparative sequence analysis of *Sordaria macrospora* and *Neurospora crassa* as a means to improve genome annotation. Fungal Genet Biol.

[CR59] Nowrousian M, Ringelberg C, Dunlap JC, Loros JJ, Kück U (2005). Cross-species microarray hybridization to identify developmentally regulated genes in the filamentous fungus *Sordaria macrospora*. Mol Gen Genomics.

[CR60] Nowrousian M, Frank S, Koers S, Strauch P, Weitner T, Ringelberg C, Dunlap JC, Loros JJ, Kück U (2007). The novel ER membrane protein PRO41 is essential for sexual development in the filamentous fungus *Sordaria macrospora*. Mol Microbiol.

[CR61] Nowrousian M, Stajich JE, Chu M, Engh I, Espagne E, Halliday K, Kamerewerd J, Kempken F, Knab B, Kuo HC, Osiewacz HD, Pöggeler S, Read ND, Seiler S, Smith KM, Zickler D, Kück U, Freitag M (2010). *De novo* assembly of a 40 Mb eukaryotic genome from short sequence reads: *Sordaria macrospora*, a model organism for fungal morphogenesis. PLoS Genet.

[CR62] Nowrousian M, Teichert I, Masloff S, Kück U (2012). Whole-genome sequencing of *Sordaria macrospora* mutants identifies developmental genes. G3 (Bethesda).

[CR63] Ohsumi Y (2001). Molecular dissection of autophagy: two ubiquitin-like systems. Nat Rev Mol Cell Biol.

[CR64] Ohsumi Y (2014). Historical landmarks of autophagy research. Cell Res.

[CR65] Pöggeler S, Kück U (2004). A WD40 repeat protein regulates fungal cell differentiation and can be replaced functionally by the mammalian homologue striatin. Eukaryot Cell.

[CR66] Pöggeler S, Kück U (2006). Highly efficient generation of signal transduction knockout mutants using a fungal strain deficient in the mammalian *ku70* ortholog. Gene.

[CR67] Pöggeler S, Nowrousian M, Jacobsen S, Kück U (1997). An efficient procedure to isolate fungal genes from an indexed cosmid library. J Microbiol Meth.

[CR68] Pöggeler S, Masloff S, Hoff B, Mayrhofer S, Kück U (2003). Versatile EGFP reporter plasmids for cellular localization of recombinant gene products in filamentous fungi. Curr Genet.

[CR69] Pöggeler S, Nowrousian M, Ringelberg C, Loros JJ, Dunlap JC, Kück U (2006). Microarray and real time PCR analyses reveal mating type-dependent gene expression in a homothallic fungus. Mol Gen Genomics.

[CR70] Pöggeler S, Nowrousian M, Teichert I, Beier A, Kück U, Anke T, Schüffler A (2018). Fruiting body development in ascomycetes. The mycota XV, physiology and genetics.

[CR71] Radchenko D, Teichert I, Pöggeler S, Kück U (2018). A Hippo pathway-related GCK controls both sexual and vegetative developmental processes in the fungus *Sordaria macrospora*. Genetics.

[CR72] Rech C, Engh I, Kück U (2007). Detection of hyphal fusion in filamentous fungi using differently fluorescene-labeled histones. Curr Genet.

[CR73] Reschka EJ, Nordzieke S, Valerius O, Braus GH, Pöggeler S (2018). A novel STRIPAK complex component mediates hyphal fusion and fruiting-body development in filamentous fungi. Mol Microbiol.

[CR74] Rogov V, Dötsch V, Johansen T, Kirkin V (2014). Interactions between autophagy receptors and ubiquitin-like proteins form the molecular basis for selective autophagy. Mol Cell.

[CR75] Schindler D, Nowrousian M (2014). The polyketide synthase gene *pks4* is essential for sexual development and regulates fruiting body morphology in *Sordaria macrospora*. Fungal Genet Biol.

[CR76] Schumacher DI, Lütkenhaus R, Altegoer F, Teichert I, Kück U, Nowrousian M (2018). The transcription factor PRO44 and the histone chaperone ASF1 regulate distinct aspects of multicellular development in the filamentous fungus *Sordaria macrospora*. BMC Genet.

[CR77] Scott B, Eaton CJ (2008). Role of reactive oxygen species in fungal cellular differentiations. Curr Opin Microbiol.

[CR78] Siegmund U, Marschall R, Tudzynski P (2015). BcNoxD, a putative ER protein, is a new component of the NADPH oxidase complex in *Botrytis cinerea*. Mol Microbiol.

[CR79] Spedale G, Timmers HT, Pijnappel WW (2012). ATAC-king the complexity of SAGA during evolution. Genes Dev.

[CR80] Steffens EK, Becker K, Krevet S, Teichert I, Kück U (2016). Transcription factor PRO1 targets genes encoding conserved components of fungal developmental signaling pathways. Mol Microbiol.

[CR81] Svenning S, Lamark T, Krause K, Johansen T (2011). Plant NBR1 is a selective autophagy substrate and a functional hybrid of the mammalian autophagic adapters NBR1 and p62/SQSTM1. Autophagy.

[CR82] Teichert I (2018). Adenosine to inosine mRNA editing in fungi and how it may relate to fungal pathogenesis. PLoS Pathog.

[CR83] Teichert I, Wolff G, Kück U, Nowrousian M (2012). Combining laser microdissection and RNA-seq to chart the transcriptional landscape of fungal development. BMC Genomics.

[CR84] Teichert I, Nowrousian M, Pöggeler S, Kück U (2014). The filamentous fungus *Sordaria macrospora* as a genetic model to study fruiting body development. Adv Genet.

[CR85] Teichert I, Steffens EK, Schnaß N, Fränzel B, Krisp C, Wolters DA, Kück U (2014). PRO40 is a scaffold protein of the cell wall integrity pathway, linking the MAP kinase module to the upstream activator protein kinase C. PLoS Genet.

[CR86] Teichert I, Dahlmann T, Kück U, Nowrousian M (2017). RNA editing during sexual development occurs in distantly related filamentous ascomycetes. Genome Biol Evol.

[CR87] Teichert I, Lutomski M, Märker R, Nowrousian M, Kück U (2017). New insights from an old mutant: SPADIX4 governs fruiting body development but not hyphal fusion in *Sordaria macrospora*. Mol Gen Genomics.

[CR88] Traeger S, Nowrousian M (2015). Functional analysis of developmentally regulated genes *chs7* and *sec22* in the ascomycete *Sordaria macrospora*. G3 (Bethesda).

[CR89] Traeger S, Altegoer F, Freitag M, Gabaldon T, Kempken F, Kumar A, Marcet-Houben M, Pöggeler S, Stajich JE, Nowrousian M (2013). The genome and development-dependent transcriptomes of *Pyronema confluens*: a window into fungal evolution. PLoS Genet.

[CR90] Voigt O, Pöggeler S (2013). Autophagy genes *Smatg8* and *Smatg4* are required for fruiting-body development, vegetative growth and ascospore germination in the filamentous ascomycete *Sordaria macrospora*. Autophagy.

[CR91] Voigt O, Pöggeler S (2013). Self-eating to grow and kill: autophagy in filamentous ascomycetes. Appl Microbiol Biotechnol.

[CR92] Voigt O, Herzog B, Jakobshagen A, Pöggeler S (2013). bZIP transcription factor SmJLB1 regulates autophagy-related genes *Smatg8* and *Smatg4* and is required for fruiting-body development and vegetative growth in *Sordaria macrospora*. Fungal Genet Biol.

[CR93] Voigt O, Herzog B, Jakobshagen A, Pöggeler S (2014). Autophagic kinases SmVPS34 and SmVPS15 are required for viability in the filamentous ascomycete *Sordaria macrospora*. Microbiol Res.

[CR94] Walz M, Kück U (1995). Transformation of *Sordaria macrospora* to hygromycin B resistance: characterization of transformants by electrophoretic karyotyping and tetrad analysis. Curr Genet.

[CR95] Wen X, Klionsky DJ (2016). An overview of macroautophagy in yeast. J Mol Biol.

[CR96] Werner A, Herzog B, Frey S, Pöggeler S (2016). Autophagy-associated protein SmATG12 is required for fruiting-body formation in the filamentous ascomycete *Sordaria macrospora*. PLoS One.

[CR97] Werner A, Herzog B, Voigt O, Valerius O, Braus GH, Pöggeler S (2019). NBR1 is involved in selective pexophagy in filamentous ascomycetes and can be functionally replaced by a tagged version of its human homolog. Autophagy.

[CR98] Yin X, Zhou C, Li J, Liu R, Shi B, Yuan Q, Zou S (2019). Autophagy in bone homeostasis and the onset of osteoporosis. Bone Res.

[CR99] Zhou JJ, Wang J, Cheng Y, Chi YJ, Fan B, Yu JQ, Chen Z (2013). NBR1-mediated selective autophagy targets insoluble ubiquitinated protein aggregates in plant stress responses. PLoS Genet.

[CR100] Zhu XM, Li L, Wu M, Liang S, Shi HB, Liu XH, Lin FC (2019). Current opinions on autophagy in pathogenicity of fungi. Virulence.

[CR101] Zickler D, Espagne E (2016). *Sordaria*, a model system to uncover links between meiotic pairing and recombination. Semin Cell Dev Biol.

